# Reviewing the current state of virtual reality integration in medical education - a scoping review

**DOI:** 10.1186/s12909-024-05777-5

**Published:** 2024-07-23

**Authors:** Marvin Mergen, Norbert Graf, Marcel Meyerheim

**Affiliations:** Department of Pediatric Oncology and Hematology, Faculty of Medicine, Saarland University, Building 9, Kirrberger Strasse 100, 66421 Homburg, Germany

**Keywords:** Digitalisation, Medical Education, Medical School, Medical training, Virtual reality

## Abstract

**Background:**

In medical education, new technologies like Virtual Reality (VR) are increasingly integrated to enhance digital learning. Originally used to train surgical procedures, now use cases also cover emergency scenarios and non-technical skills like clinical decision-making. This scoping review aims to provide an overview of VR in medical education, including requirements, advantages, disadvantages, as well as evaluation methods and respective study results to establish a foundation for future VR integration into medical curricula.

**Methods:**

This review follows the updated JBI methodology for scoping reviews and adheres to the respective PRISMA extension. We included reviews in English or German language from 2012 to March 2022 that examine the use of VR in education for medical and nursing students, registered nurses, and qualified physicians. Data extraction focused on medical specialties, subjects, curricula, technical/didactic requirements, evaluation methods and study outcomes as well as advantages and disadvantages of VR.

**Results:**

A total of 763 records were identified. After eligibility assessment, 69 studies were included. Nearly half of them were published between 2021 and 2022, predominantly from high-income countries. Most reviews focused on surgical training in laparoscopic and minimally invasive procedures (43.5%) and included studies with qualified physicians as participants (43.5%). Technical, didactic and organisational requirements were highlighted and evaluations covering performance time and quality, skills acquisition and validity, often showed positive outcomes. Accessibility, repeatability, cost-effectiveness, and improved skill development were reported as advantages, while financial challenges, technical limitations, lack of scientific evidence, and potential user discomfort were cited as disadvantages.

**Discussion:**

Despite a high potential of VR in medical education, there are mandatory requirements for its integration into medical curricula addressing challenges related to finances, technical limitations, and didactic aspects. The reported lack of standardised and validated guidelines for evaluating VR training must be overcome to enable high-quality evidence for VR usage in medical education. Interdisciplinary teams of software developers, AI experts, designers, medical didactics experts and end users are required to design useful VR courses. Technical issues and compromised realism can be mitigated by further technological advancements.

## Introduction

The focus of medical education is gradually shifting towards digital learning methods, with an increasing emphasis on incorporating new technologies facilitated by the rapid progress in computer science, particularly in artificial intelligence (AI). This has led to the adoption and integration of Virtual Reality (VR) as an educational tool for prospective and registered health care professionals, covering an ever-expanding range of use-cases.

### Developments of VR technology

 Based on the literature, the first ten years of the 21st century can be referred to as the “VR winter” [1], characterised by a lack of public interest in this emerging technology. However, there was continuous but limited research taking place in corporate, academic, and military research facilities worldwide. The widespread adoption of VR was hindered by the high costs involved, with hardware expenses exceeding $35,000 for a so-called head-mounted display (HMD) and over $30,000 for tracking equipment. Moreover, the fragile infrastructure posed additional challenges for its widespread usage. Around 2011, there was a renewed interest in consumer-grade VR, primarily driven by its potential for entertainment. Prominent companies like Valve, NVIDIA, and the start-up Oculus played a significant role in advancing HMD-based VR technology, transitioning it from being exclusive to technical elites in specialised labs to becoming a mainstream medium for content consumption available to the public [1].

### Immersive versus screen-based VR

By now, VR can broadly be divided into screen-based applications (as widely used in surgical simulators) and immersive solutions (wearing HMDs). While literature comparing effectiveness between these two approaches is sparse, according to Gutiérrez et al. [2], study participants benefited significantly more from immersive than screen-based training in terms of knowledge gain, which is recommended to be explored in more detail [3].

### Development of VR use cases in medical education

Having experienced its primary use case in the surgical field, VR simulations have been shown to be beneficial for robotic surgery training [4]. In addition, according to Izard et al. [5], VR tools have proven effective in providing in-depth knowledge of surgical interventions. This technology allows users to repeat all procedural steps as often as needed to accommodate individual learning progress, an approach which would be impractical in real-world settings [5].

As learning can be particularly challenging in critical situations where repeated training is not feasible due to the risk to patients’ lives, VR training deserves special attention for acquiring routine in emergency scenarios, such as cardiopulmonary resuscitation [6].

Furthermore, effective and enforced communication, as one example of non-technical skills (NTS), is gaining increasing awareness for healthcare providers. The impact of NTS in medical education and respective learning goals are growing likewise to the students’ demands to practise these skills in VR [7]. While training applications to develop NTS in VR are still underrepresented, vast developments in AI encourage growing numbers of studies with respective immersive simulations [8]. Additionally, evidence for VR simulations being beneficial for communication skills, emotional management, critical thinking, and clinical decision-making is expanding [9–11].

Between 2020–2022, the SARS-CoV-2 pandemic fuelled integration of digital solutions into medical curricula [12] which at the same time increased use cases for VR technology as demonstrated by Birrenbach et al. [13]: e.g., to train tasks such as hand disinfection, nasopharyngeal swab-taking as well as the proper wearing and removal of personal protective equipment.

### Students’ perception of VR

Overall, students’ perception of integrating VR into medical education is very positive as shown by De Ponti et al. [14] as well as a survey conducted at our medical faculty in 2022 [7]. To further enhance acceptance of VR, its deep integration into medical curricula is required [15]. Simultaneously, cost-effectiveness can be realised in the long run compared to traditional training methods that rely on cadavers, manikins or actors [16, 17].

This scoping review has been conducted in parallel to the project “medical tr.AI.ning” [18], which aims to develop an AI-based immersive VR learning platform that enables medical students to practise clinical decision-making with interactive virtual patients in realistic environments.

### Currently available reviews and gaps

During the initial search on MEDLINE (PubMed), we identified one scoping review which addresses VR in medical education as well but with a limited study cohort of undergraduate or pre-registration medical students [19]. Jiang et al. [19] focused on the technology deployment of VR tools, their features and respective study characteristics. Our scoping review goes beyond this publication by exploring additional aspects of integrating VR in medical education, not only regarding undergraduate/pre-registered medical and nursing students but also physicians and registered nurses. As an extension to Jiang et al., we also include reported evaluation methods and results as well as advantages and disadvantages of applying VR in medical education.

Another scoping review covering a similar topic was recently published by Lie et al. [20]. Nevertheless, they only included 7 papers that solely focused on immersive VR and searched other databases, such as Academic Search Elite, Education Source, or Google Scholar. Still, they presented valuable recommendations regarding the integration of VR into health professions education while referring to Carl May’s general theory of implementation [21]. Therefore, they identified 7 categories which must be considered: collaboration, availability, expenses, guidelines, technology/usability, careful design & evaluation and training [20].

Furthermore, we identified a scoping review dealing with the effects of VR in medical education published in February 2023 [22], wherein 11 out of 28 included studies focused on education. However, this study did not include reviews and registered nurses as study cohorts. Apart from reporting clinical outcomes, the main goal of their scoping review was the assessment of specific endpoints in medical training, such as knowledge, skills or confidence. While providing important insights into the positive outcomes of VR use in medical education, our study covers additional aspects, such as requirements, advantages or disadvantages of VR used for educational purposes in the medical field.

### Aim of this review

This scoping review aims to provide an overview of the current use and status of VR in medical education and to excerpt the requirements, advantages, and disadvantages associated with integrating this technology for training health care professionals. Furthermore, we assessed whether and how the use of VR technology has been evaluated and which results were derived to establish a foundation on which further VR projects can be developed, evaluated, and integrated into medical curricula.

### Review questions

This review aims to address the following questions:

- RQ1: How many reviews are from Germany, Europe, worldwide in general published in English or German language?
- RQ2: How often is VR used for training medical/nursing students, qualified physicians, registered nurses?
- RQ3: In which subjects/curricula is VR used in medical education?
- RQ4: Which technical and didactic requirements are reported for using VR in medical education?
- RQ5: Is VR evaluated in medical education? If yes, how is it evaluated and which outcomes are reported?
- RQ6: Which advantages of VR in medical education are reported?
- RQ7: Which disadvantages of VR in medical education are reported?

## Methods

The presented scoping review was conducted in accordance with the updated JBI methodology for scoping reviews [23] as well as in line with the PRISMA extension for scoping reviews (PRISMA-ScR) [24]. The corresponding protocol was developed in alignment with PRISMA-P [25] and has been made publicly available [26].

### Inclusion criteria

#### Participants

This scoping review did not include patient recruitment or public involvement itself, but we examined reviews that included studies on the use of VR in the education of medical and nursing students, registered nurses and qualified physicians.

#### Concept

In alignment with the defined research questions, reviews which cover the application of VR as a tool for health care professional education were considered for this work. As foundation, only reviews being published from 2012 until March 2022 were considered as eligible publications since they most comprehensively cover knowledge gain and developments in this research area. Open access publications and publications accessible via our institution in English as global scientific language or German as native language of the authors of this review were taken into account. Reviews without focus on medical education, e.g., the application of VR in patient treatment, as well as publications which centred Augmented / Extended / or Mixed Reality were excluded. Although this review also analyses requirements, evaluation concepts and results, advantages and disadvantages of using VR in medical education, the overarching concept is to examine how and in which medical curricula VR has been already used to date.

#### Context

Findings of this review can contribute as a foundation to development directions for VR applications, their integration into medical education and respective study designs. Considering the still high initial acquisition costs of VR hardware, differences between high- and low-income countries are likely to reveal. Regarding the joint German project “medical tr.AI.ning” [18], it is of particular interest to explore how and to what extent VR has been used in medical education yet in Germany, Europe and in comparison to the rest of the world as well as to identify respective gaps.

#### Type of sources

Resulting from a preliminary search in MEDLINE (PubMed), the literature search was narrowed down to focus only on reviews that topic corresponding studies on VR since they most appropriately summarise knowledge acquisition and developmental directions within this research field while providing scientific evidence.

### Search strategy

A three-step search strategy was established to target eligible publications according to the applied methodology [23]. After a preliminary search of MEDLINE (PubMed) which was independently conducted by two authors, search terms were compared and a consensus for search terminology and strategy was established for MEDLINE (PubMed), ScienceDirect (Elsevier), Web of Science Core Collection (Clarivate), Cochrane Library (Wiley), and JBI Evidence Synthesis (Wolter Kluwer) (see Table 1 ) and reviewed by the third author.

**Table 1:** Full search strategy in PubMed, conducted in March 2022

Search	Query	Results
**#1**	“Virtual Reality“[Mesh] OR **“**virtual realit*”[tw] OR VR[tw]	19,800 results
**#2**	“Students, Medical“[Mesh] OR “Education, Medical“[Mesh] OR “medical educat*”[tw] OR “medical teach*”[tw] OR “medical train*”[tw] OR “health professional educat*”[tw] OR “health professional teach*”[tw] OR “health professional train*”[tw] OR “medical school” [tw] OR “nursing train*”[tw] OR “nursing teach*”[tw] OR “nursing educat*” [tw] OR “physician train*”[tw] OR “physician teach*” [tw] OR “physician educat*”[tw] OR “doctor train*”[tw] OR “doctor teach*”[tw] OR “doctor educat*” [tw] OR “health care professional train*” [tw] OR “health care professional educat*”[tw]	314,100 results
**#3**	#1 AND #2	1,734 results
**#4**	#1 AND #2 Filters: Review	246 results
**#5**	#1 AND #2 Filters: Review, English	227 results
**#6**	#1 AND #2 Filters: Review, English, German	234 results
**#7**	#1 AND #2 Filters: Review, English, German, from 2012–2022	169 results

### Study/Source of evidence selection

**Fig. 1:**
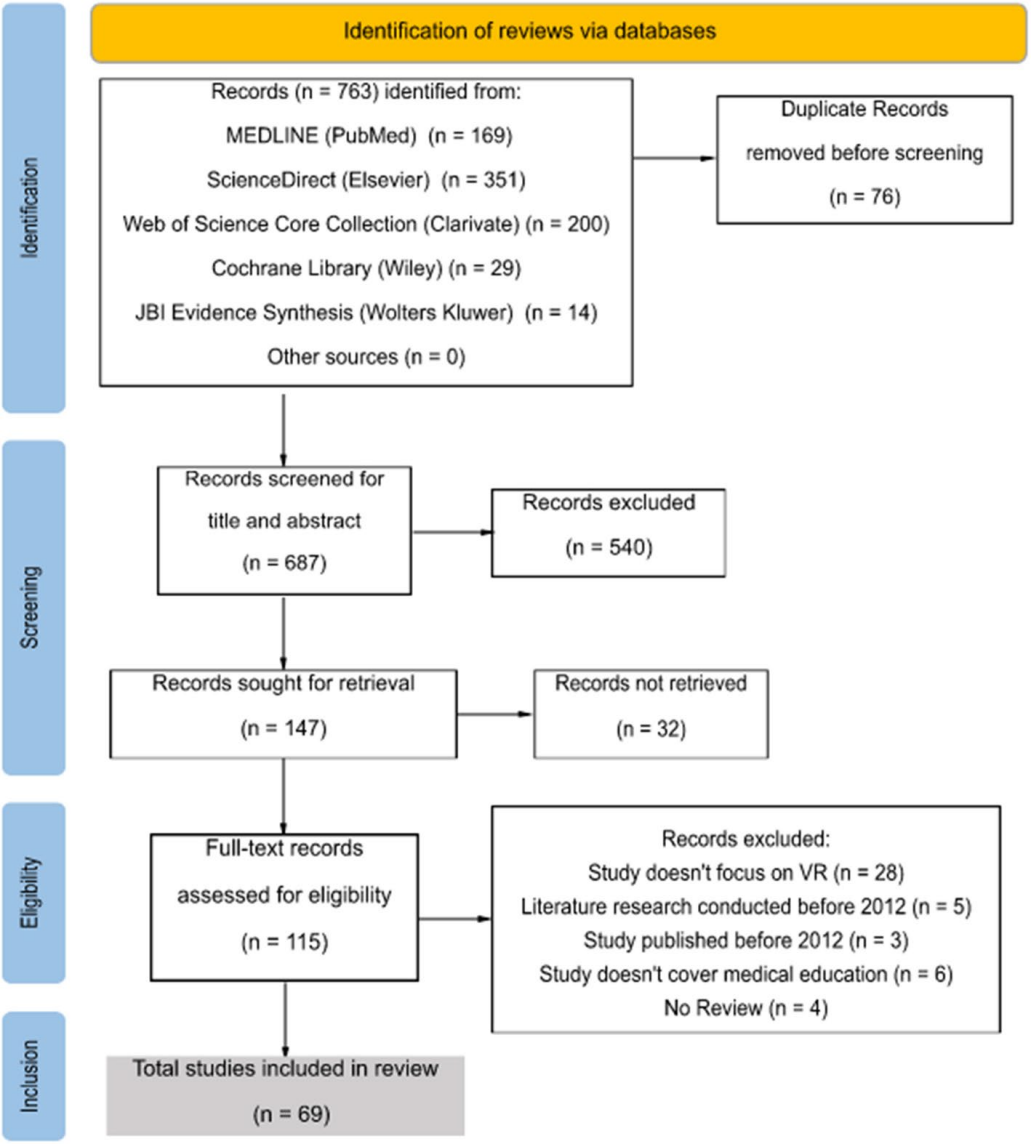
PRISMA2020-flow diagram of search and study selection process. VR: virtual reality

Following the database search, all identified records were uploaded into Mendeley Desktop (version 1.19.8, 2020) and duplicates were removed. After a pilot test, titles and abstracts were screened by two independent reviewers following the established inclusion criteria. Full texts of potentially eligible studies were retrieved, imported to the JBI System for the Unified Management, Assessment and Review of Information (JBI SUMARI) [27] and independently screened by the two reviewers following the inclusion criteria while documenting reasons for exclusion. Any arising disagreements during the whole screening process were resolved through discussion and consensus or consulting the third reviewer. Results of the evidence selection are presented as PRISMA2020-flow diagram (see Fig. 1) [28].

### Data extraction

After full text screening and a data extraction pilot test, information relevant for the research questions were independently retrieved from included reviews by two reviewers using a spreadsheet with a focus on details about the medical specialty, subject and curriculum in which VR was used, details on technical/didactic requirements, evaluation methodology and outcomes, as well as advantages and disadvantages of VR in medical education. The revised draft extraction form is provided in Table 2. Any disagreements arising between the reviewers were resolved through discussions and consensus or consulting the third reviewer.

**Table 2:** Final data extraction form. *=fields which were added or modified in comparison to the originally published protocol [26]

Category	Type of Data
Bibliographic information	1. DOI* 2. Title* 3. First author* 4. Year of publication 5. Country of origin 6. Objective(s) of the review* 7. Type of review (systematic review, scoping review, etc.)* 8. Number of studies included in review*
Research questions	1. Subject/Curriculum 2. Population (physicians, nurses, medical students, etc.)* 3. Examples of application* 4. VR modality (immersive, screen-based, etc.)* 5. Requirements (technical/didactical/organisational)* 6. Evaluation performed or discussed (yes/no/partially)* 6.1 If yes, how and what has been evaluated? * 6.2 Results of evaluation* 7. Advantages 8. Disadvantages 7. Comments/Remarks*

The JBI SUMARI data extraction tool was used. The previously drafted version of the published protocol [26] was modified at the beginning of the data extraction process to improve lucidity and provide more information regarding the research questions. Additional/modified fields are marked with an asterisk

### Data analysis and presentation

Findings are presented following the PRISMA-ScR checklist [24]. Evidence is primarily presented in tabular form. A narrative summary accompanies the tabulated and/or charted results and outlines how the results are linked to the research questions.

## Results

### Included reviews

As an outcome of the applied search strategy from March 3 to March 10, 2022, a total of 763 records were identified in literature databases: 169 from MEDLINE (Pubmed), 351 from ScienceDirect (Elsevier), 200 from Web of Science Core Collection (Clarivate), 29 from Cochrane Library (Wiley), and 14 from JBI Evidence Synthesis (Wolter Kluwer). The full PRISMA2020-flow diagram is displayed in Fig. 1. After removing 76 duplicates, 687 records were screened based on their title and abstract, resulting in 540 exclusions. Out of the remaining 147 records sought for retrieval, 32 articles with restricted access were excluded since our institution did not grant access to them. From the 115 full-text records assessed for eligibility, a total of 69 studies were finally included in the review. Records were excluded according to the following criteria: no focus on VR-technology (*n* = 28), literature research of the respective review conducted before 2012 (*n* = 5), review published before 2012 (*n* = 3), medical education not covered (*n* = 6), or not being a review (*n* = 4).

### Characteristics of included reviews (RQ1)

Table 3 contains an overview of the characteristics of the included reviews regarding country of origin according to the first author as well as language, year of publication, type of review, number of included studies and which types of VR were investigated:

**Table 3:** Geographic and basic characteristics of the included reviews. Linked to RQ1.

	Number of reviews	% of 69 included reviews
COUNTRY OF ORIGIN		
**Europe**	**24**	**34.8**
United Kingdom	12	17.4
France	3	4.3
Germany	2	2.9
Netherlands	2	2.9
Belgium	1	1.4
Denmark	1	1.4
Greece	1	1.4
Ireland	1	1.4
Switzerland	1	1.4
** North America**	**21**	**30.4**
USA	15	21.7
Canada	6	8.7
** Asia**	**14**	**20.3**
China	4	5.8
Singapore	3	4.3
Iran	2	2.9
Pakistan	2	2.9
Cyprus (Turkey)	1	1.4
Malaysia	1	1.4
Turkey	1	1.4
** South America**	**5**	**7.2**
Brazil	5	7.2
** Australia**	**5**	**7.2**
**LANGUAGE**		
English	67	97.1
German	2	2.9
**YEAR OF PUBLICATION**		
2012–2014	5	7.2
2015–2017	12	17.4
2018–2020	24	34.8
2020–2022	28	40.6
**TYPE OF REVIEW**		
Not specified	30	43.5
Systematic	27	39.1
Meta-analysis	4	5.8
Narrative	3	4.3
Integrative	2	2.9
Consensus conference paper	1	1.4
Current Status and Perspectives	1	1.4
Scoping	1	1.4
**INCLUDED STUDIES**		
10	8	11.6
10–19	11	15.9
20–29	11	15.9
30–39	6	8.7
40–49	2	2.9
50	9	13.0
**TYPE OF VR**		
Screen-based	26	37.7
Not specified	18	26.1
Immersive and screen-based	14	20.3
Immersive	11	15.9

#### Country of origin

Out of the 69 included reviews, 24 (34.8%) were from Europe, 21 (30.4%) from North America, 14 (20.3%) from Asia, 5 (7.2%) each from South America and Australia, respectively. No reviews originated from Africa. Among European countries, the United Kingdom was most prevalent (*n* = 12) followed by France (*n* = 3). Germany was represented with 2 out of 69 included publications. Among North America, the USA was predominant (*n* = 15), followed by Canada (*n* = 6). 5 publications from Brazil were the only representatives of South America. Among Asia, 4 reviews were included from China as well as 3 from Singapore.

#### Language

Considering the language of included publications, English was almost exclusively predominant with 67 reviews (97.1%). Only 2 (2.9%) German-language publications were included in this review.

#### Year of publication

**Fig. 2:**
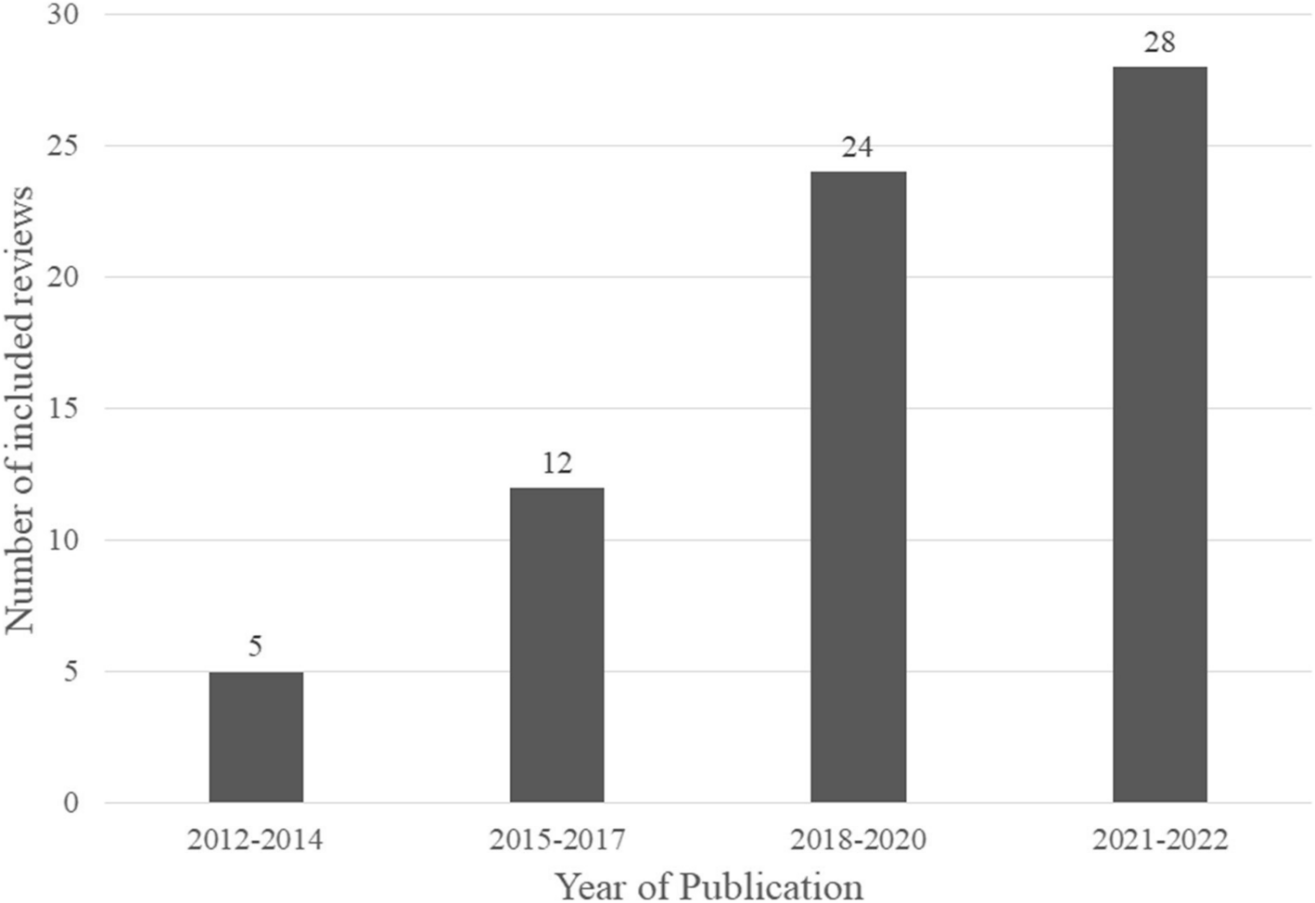
Histogram of the year of publication regarding the 69 included reviews

Regarding the number of included reviews and their publication date, there is a clear increasing trend over time: while only 5 out of 69 (7.2%) included articles were published between 2012 and 2014, 12 reviews (17.4%) were published between 2015 and 2017, 24 reviews (34.8%) between 2018 and 2020 and nearly half, i.e., 28 out of 69 included articles (40.6%), published between 2021 and the time point of our literature research in March 2022 (Fig. 2).

#### Type of review

With 30 out of 69 included articles, nearly half (43.5%) were associated by their authors with a specific type of review or review methodology. 27 reviews (39.1%) were classified as systematic review, followed by 4 meta-analysis papers (5.8%) and 3 narrative reviews (4.3%).

#### Number of included studies

Similar to the non-specification of the review type, 22 out of 69 (31.9%) included reviews have not explicitly stated the number of included studies. While 8 articles (11.6%) included either less than 10 studies or between 30 and 49 studies respectively, 9 (13.0%) included more than 50 studies on VR in medical education. The remaining 22 reviews (31.8%) included between 10 and 29 studies.

#### Type of VR

Most reviews were purely focused on studies with screen-based VR applications (*n* = 26, 37.7%), while 11 (15.9%) covered only studies on immersive forms of VR, e.g., using HMDs. 14 out of 69 (20.3%) reviews included studies on both screen-based and immersive forms of VR, while 18 (26.1%) provided no specifications on the VR modality examined in the included studies.

### Study participants (RQ2)

We found different study subject populations among the studies reported by the reviews (see Table 4): 30 out of 69 (43.5%) reviews focused on studies with qualified physicians only, while 9 reviews (13.0%) addressed medical students only. Exclusively registered nurses were part of studies of 4 reviews (5.8%), while 5 (7.2%) did not explicitly specify which type of participants were included in the considered studies. The remaining reviews focused on studies addressing combinations of participants, such as medical students and qualified physicians (*n* = 8, 11.6%), medical and nursing students (*n* = 2, 2.9%) or all previously mentioned groups of participants (medical students, nursing students, qualified physicians and registered nurses) in 11 out of 69 (15.9%) reviews.

**Table 4:** Summary of groups of participants in the studies included in the reviews. “All groups” refers to medical and nursing students as well as physicians and nurses. Linked to RQ2.

	Number of reviews	% of 69 included reviews
STUDY PARTICIPANTS		
Qualified physicians	30	43.5
All groups	11	15.9
Medical students	9	13.0
Medical students and qualified physicians	8	11.6
Not specified	5	7.2
Registered Nurses	4	5.8
Medical and nursing students	2	2.9

### Subjects/Curricula (RQ3)

**Fig. 3:**
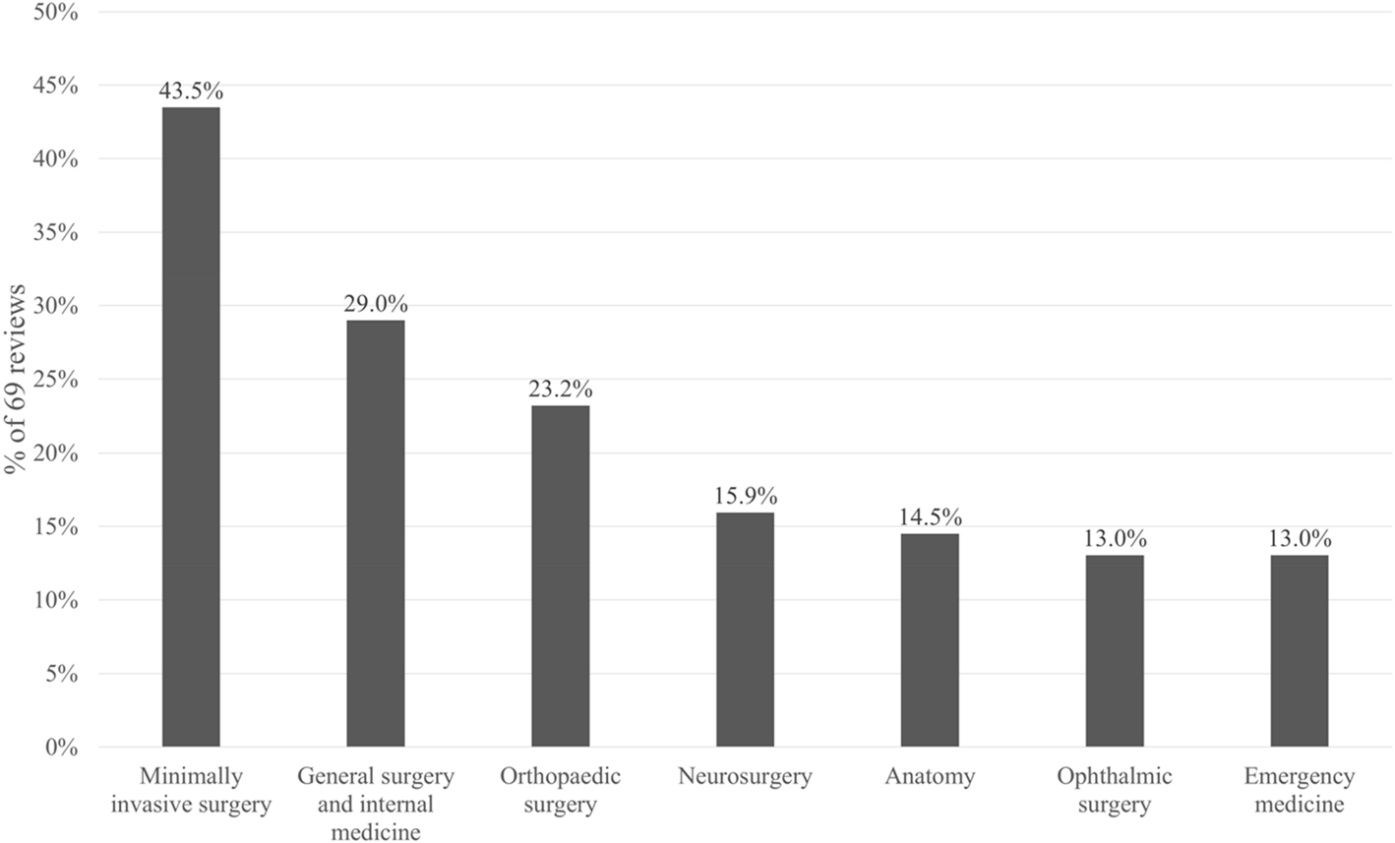
Most prevalent subjects and specialties covered by the 69 included reviews. Percentages do not add up to 100 due to overlaps by reviews covering more than one subject or specialty

The included reviews covered a broad range of subjects, specialties and skills of medical curricula regarding VR applications (see Fig. 3; Table 5). 46 out of 69 (66.7%) reviews addressed more than one subject or specialty while 21 reviews (30.4%) exclusively focused on one of them. In 2 reviews (2.9%), the outlined characteristics of the included studies did not provide clear information on the considered medical subjects.

**Table 5:** Distribution of subjects and specialties among included reviews. Percentages do not add up to 100 due to overlaps by reviews covering more than one subject or specialty. Linked to RQ3.

Subjects/Curricula	Number of Reviews	% of 69	Review References
SURGERY			
Minimally invasive	30	43.5	[19, 29, 30, 32, 34–39, 42–44, 47, 59, 65, 66, 68, 70, 73, 76, 84, 92, 96–102]
General surgery and internal medicine	20	29.0	[19, 32, 34, 35, 38, 41, 43, 44, 59, 68, 73, 76, 84, 96–98, 101–104]
Orthopaedic	16	23.2	[19, 29, 30, 34, 36, 37, 39, 41, 42, 46, 47, 59, 70, 92, 99, 104]
Neurological	11	15.9	[19, 34, 41, 59, 71, 73, 86, 94, 96, 105, 106]
Ophthalmic	9	13.0	[19, 40, 59, 63, 69, 103, 105, 107, 108]
Cardiothoracic	5	7.2	[34, 65, 92, 109, 110]
Otorhinolaryngologic	5	7.2	[19, 34, 59, 60, 92]
Vascular	4	5.8	[19, 59, 109, 111]
Urologic	4	5.8	[34, 59, 96, 100]
Dental	2	2.9	[59, 73, 112]
Plastic	1	1.4	[112]
Gynaecologic	1	1.4	[96]
Paediatric	1	1.4	[59]
Interventional radiology	1	1.4	[66]
**NON-SURGICAL**			
Emergency	9	13.0	[11, 19, 34, 44, 48, 61, 72, 73, 92]
Paediatrics	5	7.2	[19, 64, 75, 92, 113]
Urology	5	7.2	[11, 64, 73, 75, 92]
Psychiatry	4	5.8	[44, 45, 58, 92]
Pulmonology	4	5.8	[72, 75, 77, 92]
Nursing	3	4.3	[55, 75, 89]
Dentistry	2	2.9	[92, 114]
Radiology	2	2.9	[19, 59]
Neurology	2	2.9	[72, 92]
Dermatology	1	1.4	[72]
Geriatrics	1	1.4	[73]
Pathology	1	1.4	[73]
General medicine	1	1.4	[11]
Neonatology	1	1.4	[61]
Gastro	1	1.4	[92]
**PRECLINICAL**			
Anatomy	10	14.5	[19, 44, 50, 72, 73, 85, 92, 105, 115, 116]
Physiology	1	1.4	[116]
Psychology	1	1.4	[49]
**INTERDISCIPLINARY**			
Basic clinical skills	7	10.1	[19, 55, 64, 72, 75, 89, 92]
Communication	4	5.8	[11, 19, 72, 73]
Non-technical skills	2	2.9	[11, 34]
Pharmaceutical education	2	2.9	[50, 64]
Decontamination	2	2.9	[64, 75]
Not specified	2	2.9	[3, 117]
History taking	1	1.4	[72]
Surgery planning	1	1.4	[105]
Disaster management	1	1.4	[64]

#### Surgical specialties

Predominantly, studies within the scope of VR for training in surgery and surgical specialties were discussed by the included reviews with minimally invasive types of surgery (e.g., laparoscopic, endoscopic) being the subject covered by most publications (*n* = 30, 43.5%). This type of surgery is often associated with general surgery and internal medicine interventions (*n* = 20, 29.0%), orthopaedics (*n* = 16, 23.2%) and neurosurgery (*n* = 11, 15.9%). Additionally, the last group includes VR applications for pre-operative planning as well. Overall, 14 specialties related to surgery were represented by the included reviews. Depending on the specialty and procedure, applications are not covering the whole spectrum of possibilities, e.g., simulators for hip surgery or replacement were reported to lag behind other orthopaedic procedures such as knee and shoulder arthroscopy [29, 30].

#### Non-surgical specialties

Regarding non-surgical clinical specialties, emergency medicine as well as associated scenarios were most prevalent (*n* = 9, 13.0%) followed by paediatrics and urology, each represented by 5 reviews (7.2%). In total, 15 clinical specialties with no focus on surgery were covered by the included reviews besides 3 preclinical subjects, i.e., anatomy (*n* = 10, 14.5%), physiology and psychology (each *n* = 1, 1.4%). Furthermore, interdisciplinary and overarching topics, such as basic clinical or NTS (cognitive, interprofessional, social, communicative) were addressed in 14 reviews (20.3%).

### Requirements (RQ4)

**Fig. 4:**
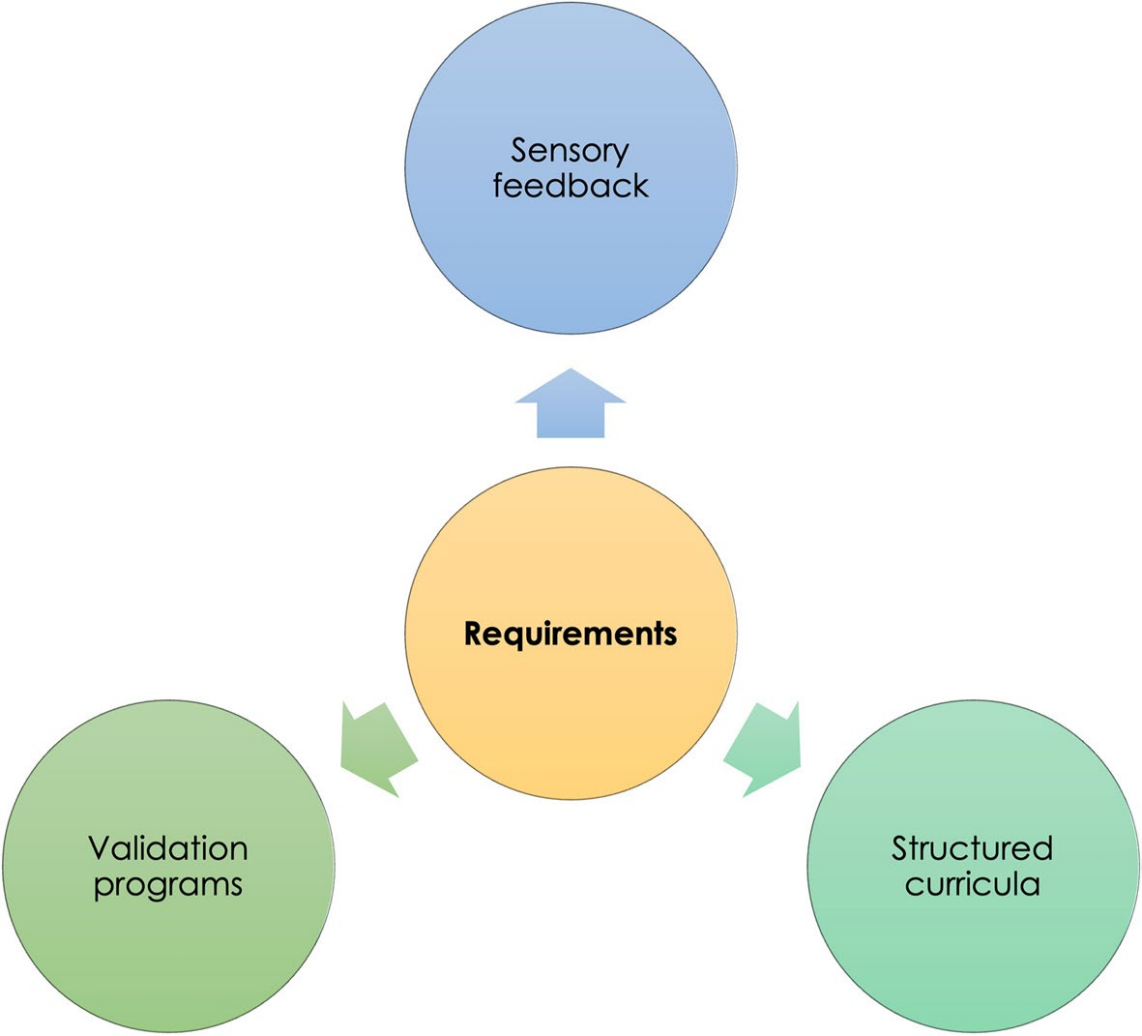
Most mentioned requirements for the development of VR applications for medical education as reported by included reviews. "Respective hardware" is not mentioned in this figure since it is a basic requirement that was addressed in every included review.

Based on our findings considering specifications of requirements mentioned in the included reviews, they were classified according to technical, didactical and organisational requirements for the integration of VR in medical education and ordered by frequency of mention (see Fig. 4; Table 6 ). Overall, 42 out of 69 reviews (39.1%) provided information on requirements.

**Table 6:** Distribution of technical, didactical and organisational requirements reported by reviews. Linked to RQ4.

	Number of Reviews	Review References
TECHNICAL REQUIREMENTS		
Respective hardware	69	All included reviews
Sensory feedback (especially haptic)	5	[29, 38, 46, 105, 108]
Appropriate amount of realism (e.g., precise contextual factors (sizes, sounds, functionalities of instruments in OR)	4	[11, 29, 37, 38]
Facilitating installation and operation (shared VR training facilities, availability on mobile devices, shared single specific platform for assessment to avoid continuous training of developers and learners)	4	[36, 48, 61, 105]
Accurate tissue interaction (difficult with current technology)	3	[37, 38, 40]
IT skills / assistance	2	[92, 105]
Gamification	1	[84]
Converting real patient data	1	[29]
**DIDACTICAL REQUIREMENTS**		
Structured curricula (incl. skill-based instruction, clear learning objectives as part of larger curricular goals, gap analysis [which skills are not adequately addressed] before early integration)	15	[3, 29, 35, 41, 42, 84, 96–98, 100, 103, 106, 110, 111, 113]
Mastery learning	4	[77, 102, 103, 113]
Deliberate practice / Self-regulated learning / Possibility to make mistakes	3	[77, 103, 113]
Mandatory involvement	2	[41, 96]
Educational training	2	[92, 105]
Progressive learning curriculum and variation of complexity	2	[84, 113]
Feedback by instructor	2	[37, 98]
Evaluation feedback by virtual trainer (tutor mode), automatically generated evaluation feedback	2	[29, 108]
Focus on clinical care incl. crisis management, teamwork, communication + problem-based learning	2	[89, 103]
Accompany cognitive material (video/reading…)	2	[35, 98]
High risk & low volume scenarios	1	[113]
Automated evaluation process	1	[59]
Reliable and validated assessment tools	1	[110]
**ORGANISATIONAL REQUIREMENTS**		
Validation programs (usability, transfer, construct, concurrent, performance metrics)	5	[3, 29, 35, 105, 108]
Cost-effectiveness	3	[30, 36, 46]
Adequate time for training to avoid cognitive overload	2	[64, 110]
VR as supplemental resource	2	[47, 100]
User-centred design	1	[105]
Comprehensive manual	1	[105]
Interdisciplinary team for development	1	[105]

Technical requirements consider the acquisition of respective hardware to be able to use VR applications, which concerns all 69 included reviews, followed by sensory/haptic feedback (*n* = 5, 7.2%) and accurate interaction regarding tissues (*n* = 3, 4.3%).

Among the didactical requirements, a strong need for well-structured curricula with sound learning theory framework and clearly defined learning objectives was highlighted most by reviews (*n* = 15, 21.7%). The concepts of mastery learning, and deliberate practice were particularly mentioned in this context (*n* = 4, 5.8% and *n* = 3, 4.3%).

Organisational aspects comprise the development of validation programs for VR training applications (considering useability, different validity levels [31], etc.) (*n* = 5, 7.2%) and evaluation of cost-effectiveness (*n* = 3, 4.3%). These are requirements before any large-scale integration.

### Synopsis of study evaluations (RQ5)

#### Summary of study methodologies

**Fig. 5:**
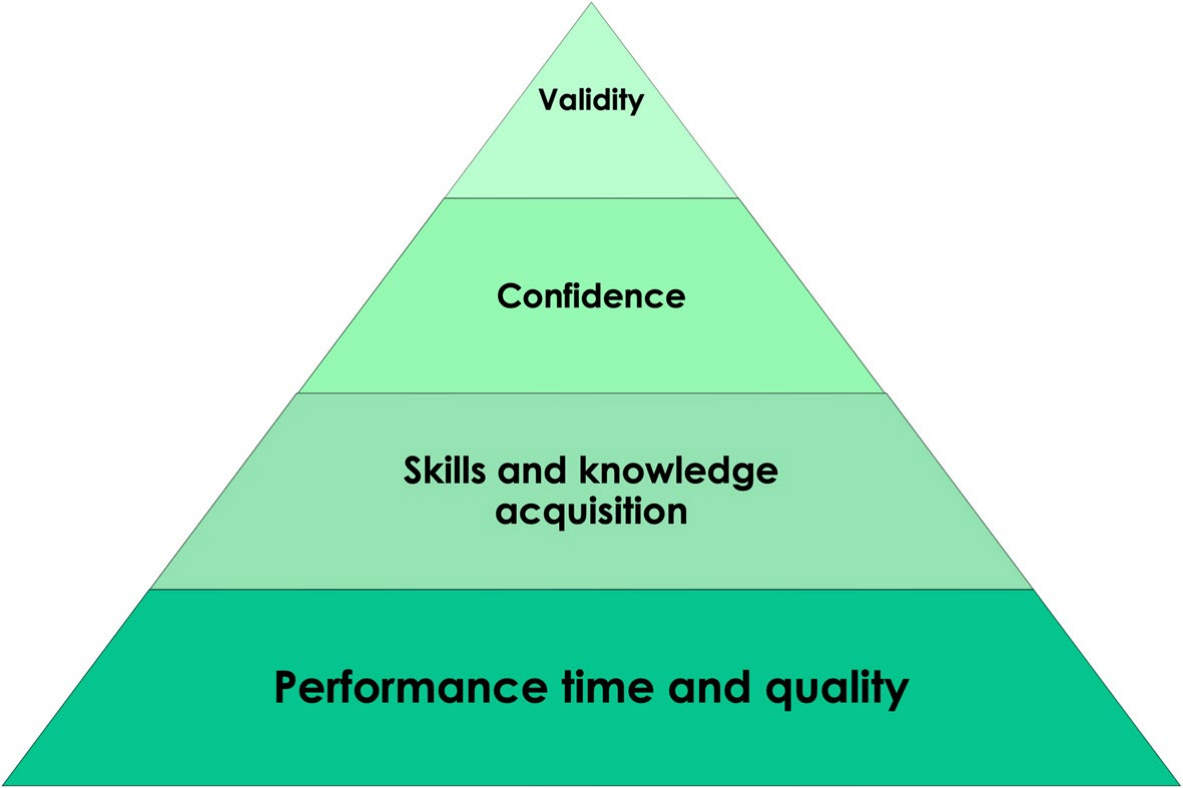
Summary of evaluation aspects reported by included reviews

Overall, 65 (94.2%) reviews mentioned evaluation aspects at least for some of their included studies (see summary in Fig. 5).

In line with the above illustrated focus on surgical procedures, evaluation results reported in most reviews (*n* = 40, 57.9%) addressed performance time and quality (e.g., completion time, procedure or examinations scores, complication rates, error rates during procedures). Results on skills and knowledge acquisition were described in 27 reviews (39.1%).

Evaluations on different levels of validity [31] were presented by 25 out of 69 reviews (36.2%). Construct validity, which questions the ability to discriminate between novice and experts, was mentioned in 14 out of 69 studies (20.3%), followed by transfer validity (*n* = 13, 18.8%), which describes the correlation between simulation- and actual real performance. Face validity, defined as the appearance of a test being appropriate, is covered in 3 reviews (4.3%). Concurrent validity, which evaluates the relationship to outcomes of another instrument purporting to measure the same construct, and content validity, covering the appropriateness of the contents of test items, are mentioned in one review each (1.4%).

Some reviews also discussed more subjective concepts, such as confidence, anxiety, satisfaction, attitude, or self-efficacy (*n* = 18, 26.1%) as well as learning curves (*n* = 9, 13.0%). Furthermore, patient outcomes (*n* = 6, 8.7%) and cost-effectiveness (*n* = 3, 4.3%) were investigated.

To evaluate the use of VR in medical education, mainly comparisons to traditional methods, such as mannequins, cadaver, desktop learning, written or video instructions, or self-study were reported in 11 reviews (15.9%). Moreover, knowledge assessment (*n* = 11, 15.9%) and quasi-experimental studies (pretest vs. posttest; *n* = 7, 10.1%) were considered. For the assessment of subjective outcomes, surveys or questionnaires were used (*n* = 6, 8.7%) besides analysing automatic quantitative metrics of simulators (*n* = 5, 7.2%).

#### Summary of study results

**Fig. 6:**
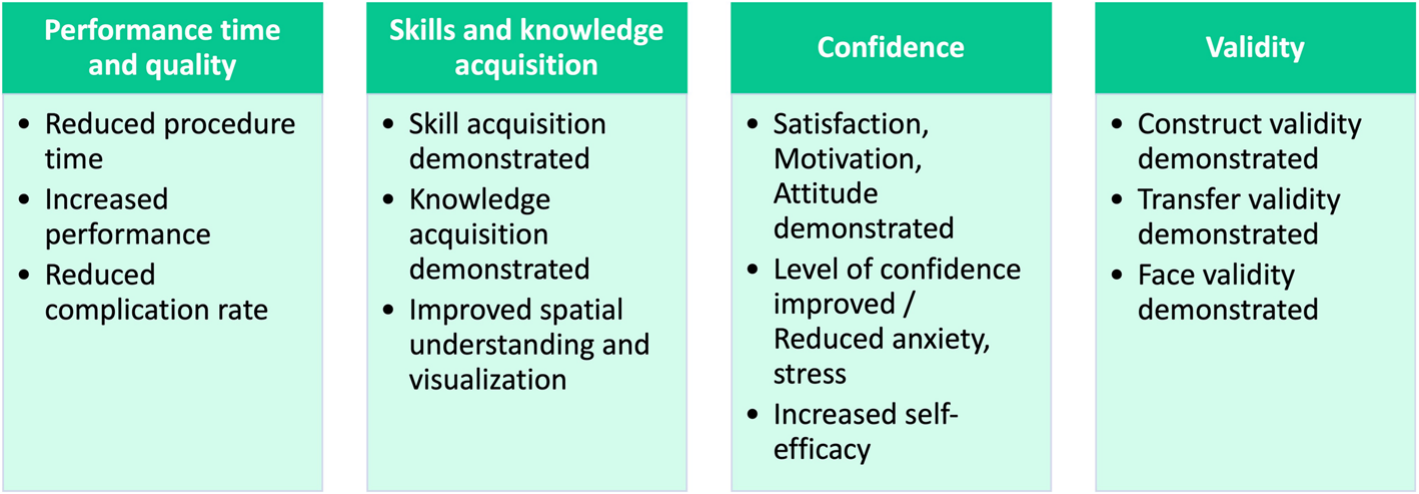
Summary of crucial evaluation results as reported by included reviews

Among the included reviews, most prevalent were reviews which included at least some information derived from the included studies’ evaluations (*n* = 65, 94.2%). Only 4 reviews (5.8%) did not present explicit details of study results. In the following, the term “mixed results” is defined by the fact that at least one of the included studies in a review did not argue in favour of VR based on comparisons regarding the analysed aspect (see summary in Fig. 6; Table 7 ).

**Table 7:** Summary of study results as reported by included reviews. Linked to RQ5.

SUMMARY OF STUDY RESULTS	Number of Reviews	Review References
PERFORMANCE TIME AND QUALITY		
Reduced procedure/performance time	20	[29, 35, 36, 39, 41, 47, 60, 66, 75, 76, 84, 92, 96, 99, 100, 102, 103, 107, 108, 110]
Reduced procedure/performance time: mixed results	6	[32, 42, 44, 69, 101, 104]
Increased procedure/examination score	10	[35, 37, 39, 42, 60, 84, 85, 92, 110, 117]
Increased procedure/examination score: mixed results	5	[32, 44, 46, 47, 69]
Increased performance	12	[46, 47, 64, 76, 89, 98, 100, 101, 105, 107, 108, 111]
Increased performance: mixed results	3	[59, 102, 104]
Reduced error rates during procedures	11	[35, 46, 63, 73, 96, 99, 101, 103, 104, 115, 117]
Reduced complication rates	8	[39–41, 66, 98, 100, 107, 109]
Reduced complication rates: mixed results	1	[69]
Significant correlation between performance in simulation and mean OSACSS scores	2	[40, 63]
**SKILLS AND KNOWLEDGE ACQUISITION**		
Skill acquisition demonstrated	11	[11, 30, 41, 45, 47, 58, 94, 99, 109, 112, 113]
Skill acquisition: mixed results	6	[3, 35, 55, 64, 75, 104]
Knowledge acquisition demonstrated	10	[3, 11, 45, 58, 60, 72, 75, 86, 94, 113]
Knowledge acquisition: mixed results	5	[47, 55, 64, 73, 116]
Improved spatial understanding and visualisation	6	[43, 44, 60, 86, 104, 115]
Less guidance needed after simulation	2	[47, 98]
Increased empathy	1	[49]
**DIFFERENT LEVELS OF VALIDITY**		
Face validity demonstrated	3	[43, 71, 111]
Content validity demonstrated	1	[71]
Construct validity demonstrated	14	[29, 36, 37, 42, 43, 47, 63, 71, 77, 97, 99, 102, 111, 112]
Concurrent validity demonstrated	1	[36]
Transfer validity demonstrated	11	[11, 35, 37, 41, 46, 64, 66, 96, 97, 99, 105]
Transfer validity: mixed results	2	[30, 70]
Evidence of complete validation is poor	1	[68]
**SATISFACTION, MOTIVATION, ATTITUDE, CONFIDENCE, SELF-EFFICACY**		
Satisfaction / Motivation / Attitude demonstrated	13	[11, 35, 45, 49, 50, 61, 72, 73, 85, 86, 89, 115, 117]
Satisfaction / Motivation /Attitude: mixed results	4	[3, 55, 64, 75]
Level of confidence improved / Reduced anxiety or stress	2	[115, 117]
Level of confidence improved / Reduced anxiety or stress: mixed results	4	[3, 55, 64, 75]
Increased self-efficacy	2	[64, 107]
**LEARNING CURVES**		
Higher improvement among novices than experts	4	[35, 37, 63, 77]
Longer learning curve in comparison to other learning methods	2	[50, 64]
Long-term retention demonstrated	2	[42, 86]
Long-term retention: mixed results	2	[44, 64]
**PATIENT OUTCOMES**		
Improved patient care quality / outcomes	6	[45, 66, 73, 84, 94, 98]
**COST-EFFECTIVENESS**		
Cost-effectiveness demonstrated	2	[3, 46]
Cost-effectiveness: lack of evidence	1	[30]

Considering performance time and quality, reduced procedure or performance time after VR simulation was outlined by 20 out of 40 reviews (50.0%), 6 reviews (15.0%) described ambiguous findings considering this aspect. 10 reviews (25.0%) detailed increased procedure or examination scores, whereas 5 (12.5%) reported mixed results. For example, Guedes et al. [32] reported that increased examination scores were observed considering basic minimally invasive surgery tasks comparing VR and box trainer groups while no difference could be detected considering other outcomes such as task completion time. The same pattern of unambiguous versus mixed results emerges regarding increased performance (12 vs. 3 reviews) and reduced complication rates (8 reviews vs. 1 review). Increased performance also implied team performance in the case of 3 reviews. Reduced error rates during procedures were described without exceptions in 11 reviews (27.5%) and a significant correlation between performance in surgical VR simulation and mean OSACSS (= Objective Structured Assessment of Cataract Surgical Skill) scores [33] was reported by 2 reviews (5.0%).

While skill acquisition after VR simulation was reported by 11 out of 27 reviews (40.7%) which contained results on skills and knowledge acquisition, 6 (22.2%) presented ambiguous results. The same pattern emerged for knowledge acquisition (10 vs. 5 reviews). Improved spatial understanding and visualisation was reported by 6 out of 27 reviews (22.2%) and one review (3.7%) described increased empathy. Additionally, less guidance by instructors was required to successfully complete a task after simulation (*n* = 2, 7.4%).

Of the 25 reviews presenting results on different levels of validity [31], 6 (24.0%) reported on more than one level of validity. Beginning with the lowest level and ordered by increasing level of validity, face validity was reported to be demonstrated in 3 reviews (12.0%), content validity in 1 (4.0%), construct validity in 14 (56.0%) and transfer validity in 11 reviews (44.0%). Considering construct validity, 2 reviews pointed out that the coarse distinction between groups of novices and experts regarding performance works well, whereas finer distinction still needs improvement.

Contradictory results considering transfer validity were reported in 2 out of the 25 reviews (8.0%), while 1 review (4.0%) reported in general poor evidence of complete validation. Except for those 3 reviews, none of the 25 reviews reported that the tested validity could not be confirmed by the included studies.

Out of the 18 publications covering results on subjective aspects, i.e., study participants’ level of confidence, anxiety, satisfaction, attitude and/or self-efficacy, 13 (72.2%) delineated that satisfaction as well as positive motivation and attitude towards VR applications were demonstrated. In contrast, 4 out of 18 (22.2%) reported mixed results. Regarding the participants’ level of confidence and anxiety, 2 reviews (11.1%) concluded that confidence improved, and anxiety or stress reduced, whereas 4 publications (22.2%) reported mixed results. Increased self-efficacy was reported in studies covered by 2 reviews (11.1%).

Considering results on learning curves, a higher improvement could be observed among novices in comparison to experts according to 4 out of 9 reviews (44.4%). Longer learning curves were reported in comparison to other already established learning methods (*n* = 2, 22.2%). Long-term retention of knowledge was reported with positive and mixed results with equal frequency (each *n* = 2, 22.2%).

Improved patient outcome and care quality after training with VR applications were reported by all 6 reviews that included results on this aspect.

Among the 3 publications covering cost-effectiveness of VR applications, 2 reviews (66.7%) reported positive results while 1 review (33.3%) claimed a general lack of evidence regarding this topic.

### Advantages of VR (RQ6)

**Fig. 7:**
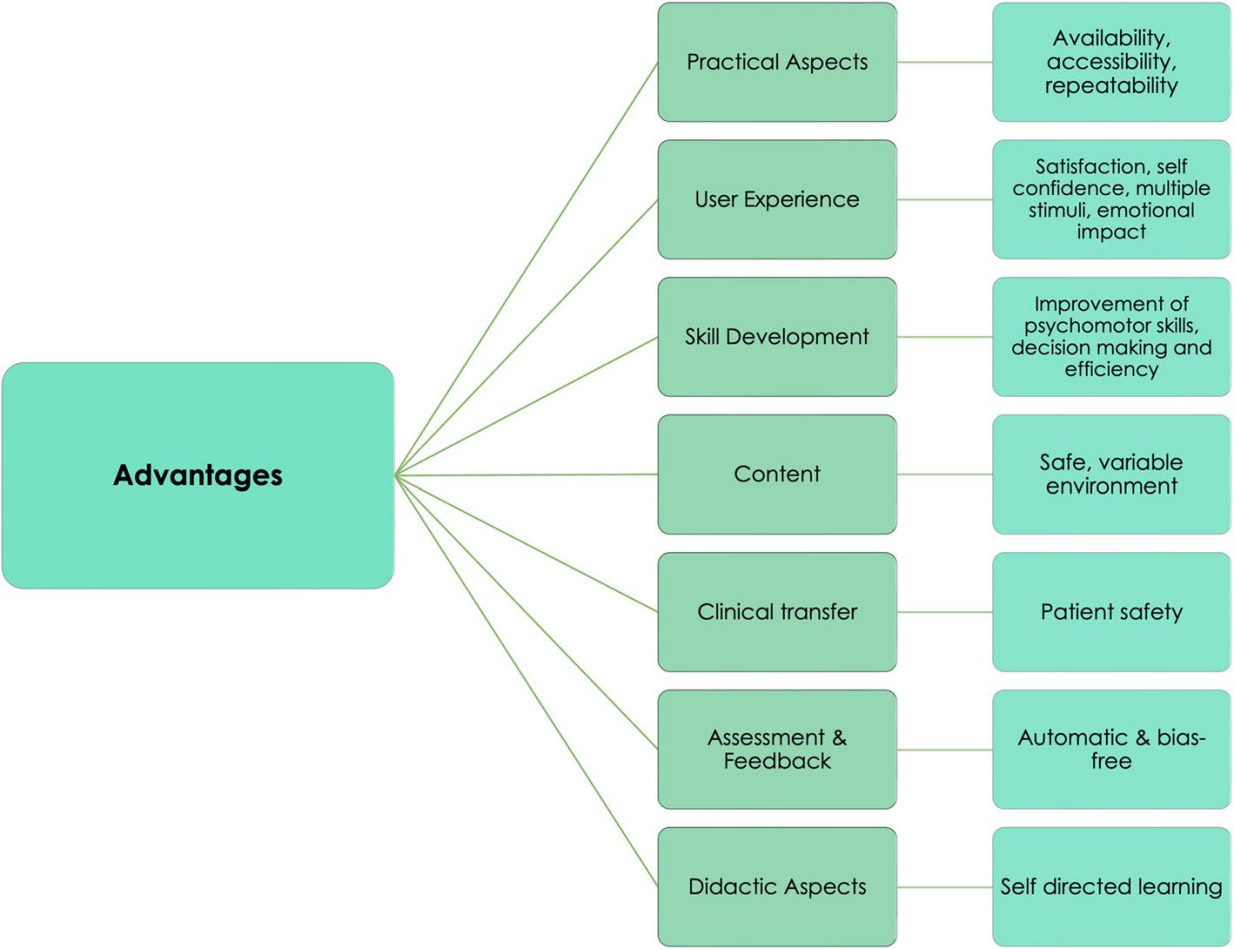
Most mentioned advantages of VR in medical education as reported in the included reviews

VR has become an increasingly popular tool in medical education. The advantages of VR in medical education that we found described in the included reviews can be organised into seven categories: (1) practical aspects, (2) content, (3) skill development, (4) clinical transfer, (5) user experience (6) assessment and feedback, and (7) didactic aspects (Fig. 7; Table 8).

**Table 8:** Advantages of VR simulations in medical education as reported by included reviews. Linked to RQ6.

REPORTED ADVANTAGES	Number of Reviews	Review References
PRACTICAL ASPECTS		
Availability/accessibility (e.g., low-cost models for low-income countries, no time restriction, easy setup, little space required)	22	[11, 19, 34, 35, 39, 44, 46–48, 58, 64, 65, 71, 72, 84, 92, 100, 102, 104, 109, 112, 113]
Repeatability	21	[11, 19, 29, 30, 34–37, 44, 46, 48, 75, 84, 86, 96, 99, 100, 102, 103, 105, 112]
Cost-effectiveness	11	[30, 32, 35, 36, 46, 48, 50, 69, 72, 113, 115]
Ethical acceptability (less use of animal and human corpses)	9	[29, 35, 37, 38, 41, 47, 97, 112, 115]
No supervision needed, reduced workload of instructors	7	[30, 32, 34, 47, 50, 76, 104]
Portability	5	[34, 40, 92, 104, 112]
No biological hazards by transmission of diseases	3	[29, 35, 112]
**USER EXPERIENCE**		
Improves satisfaction, self-confidence, educational experience, is fun	6	[64, 84, 86, 99, 108, 116]
Tactile realism by real-time haptic feedback inclusion, multimodal sensorial stimuli	6	[41, 65, 70, 96, 98, 104]
Satisfaction by user-friendliness	5	[34, 70, 86, 104, 109]
Emotional impact	2	[11, 111]
**SKILL DEVELOPMENT**		
Hands-on training, hand-eye coordination, psychomotor skills, technical skills, theory to practise	13	[11, 19, 29, 34, 46, 64, 75, 86, 92, 96, 99, 104, 108]
Competence training efficiency (reduced operating times, minimising medical errors)	12	[3, 29, 43, 46, 50, 58, 63, 69, 84, 96, 99, 104]
Improve judgement, critical thinking, decision-making, creativity, conceptual and procedural learning	8	[29, 48, 50, 61, 64, 75, 89, 96]
Training of soft skills (communication, interpersonal skills, teamwork)	7	[40, 44, 50, 61, 64, 84, 116]
Improve situational awareness, attention span	4	[40, 50, 66, 86]
**CONTENT**		
Safe, controlled environment (learn from errors, stress-free, no time constraints)	17	[29, 39, 41, 44, 47, 49, 58, 63, 64, 66, 77, 84, 89, 99, 105, 108, 115]
Variability by many use cases	13	[29, 32, 35, 41, 47, 48, 58, 65, 84, 89, 99, 100, 112]
Training of high-pressure (complex, unexpected) & low frequency scenarios	7	[11, 34, 44, 48, 89, 92, 99]
Realistic immersive environments, higher authenticity	7	[34, 37, 44, 45, 72, 84, 111]
High degree of spatial understanding and visualisation	4	[19, 44, 86, 106]
Versatile multi-user scenarios	1	[40]
**CLINICAL TRANSFER**		
Patient safety	16	[29, 30, 32, 36, 38, 47, 49, 50, 55, 63, 69, 77, 84, 89, 98, 103]
Better clinical outcome	5	[43, 66, 69, 96, 108]
Possibility of including patient specific information	4	[29, 41, 106, 111]
**ASSESSMENT AND FEEDBACK**		
Automatic, bias-free measurement and performance assessment (vision and sensor-based tracking)	11	[29, 37–39, 46–48, 92, 99, 100, 104]
Instant, embedded real-time feedback	7	[38, 48, 64, 84, 100, 104, 114]
Standardised, reproducible feedback and simulation	6	[47, 58, 98, 109, 113, 114]
Possibility of recording training data for evaluation feedback	5	[11, 29, 92, 99, 100]
**DIDACTIC ASPECTS**		
Addresses more effective self-directed / self-paced / individual/ student-centred learning and deliberate practice	8	[48, 84, 89, 92, 99, 102, 104, 115]
Experiential learning possible (e.g., dementia)	1	[49]

1. In terms of practical aspects, which represent the most frequently addressed category in our research, VR technology is reported as highly accessible and convenient, also for lower-income countries with low-cost models. Little space is required and training itself has no time restrictions (*n* = 22, 31.9%). Practising in VR is repeatable as many times as needed (*n* = 21, 30.4%), it is considered cost-effective (*n* = 11, 15.9%), and provides reduced ethical concerns compared to e.g., dissection of corpses or animals (*n* = 9, 13.0%). Moreover, depending on the training scenario, VR does not necessarily require supervision and thus reduces the workload of instructors (*n* = 7, 10.1%). 5 reviews (7.2%) mention portability as another advantage, and 3 reviews (4.3%) emphasise the absence of biological hazards, such as transmissions of diseases.
2. Focusing on content, VR offers a safe, controlled environment that allows for stress-free learning from mistakes without time constraints (*n* = 17, 24.6%). The variability of VR scenarios provides multiple use cases consisting of single basic tasks or complex procedures (*n* = 13, 18.8.%). This also facilitates to cover unexpected or low frequency scenarios as well as scenarios with high performance pressure (*n* = 7, 10.1%) in realistic immersive environments, leading to more authentic training (*n* = 7, 10.1%). Additionally, VR can help learners to develop their spatial understanding and visualisation (*n* = 4, 5.8%) and most recent applications also provide versatile multi-user scenarios that require actual teamwork in VR (*n* = 1, 1.4%).
3. Considering skill development, VR training seems to be highly effective, e.g., including psychomotor, technical, and soft skills. It facilitates hands-on training, improves hand-eye coordination, and supports the transfer from theory to practice (*n* = 13, 18.8%). High competence training efficiency, which can be assessed through reduced operating times and decreased number of medical errors, was seen in 12 reviews (17.4%). Furthermore, practising in VR allows for improved development of judgement, critical thinking, decision-making, creativity, as well as procedural/dynamical and conceptual learning (*n* = 8, 11.6%). Additionally, soft skills such as communication, interpersonal skills, and teamwork can be addressed according to 7 reviews (10.1%). 4 studies mentioned improved situational awareness and attention span (5.8%).
4. Regarding clinical transfer, 16 reviews (23.2%) emphasised patient safety when training is supported by VR simulation. 5 reviews (7.2%) referred to improved clinical outcomes, one crucial primary concern when assessing effectiveness of medical education methods. Moreover, VR can also include representation of patient-specific information, allowing for more personalised and patient-centred training and preparation (*n* = 4, 5.8%).
5. Focusing on user experience, VR training can improve satisfaction, self-confidence and the educational experience for medical students (*n* = 6, 8.7%). The inclusion of real-time haptic feedback and multimodal sensorial stimuli improves tactile realism (*n* = 6, 8.7%). Additionally, users report high levels of satisfaction with VR (*n* = 5, 7.2%), which apparently can have an emotional impact according to 2 reviews (2.9%).
6. When it comes to assessment and feedback, automatic vision- and sensor-based performance measurement allow for bias-free evaluations (*n* = 11, 15.9%). Instant real-time feedback (*n* = 7, 10.1%) as well as the possibility of recording training data (*n* = 5, 7.2%) and support efficient training in a standardised and reproducible manner (*n* = 6, 8.7%).
7. In terms of didactic aspects, VR training addresses self-directed, self-paced, and student-centred learning and deliberate practice, which is seen to be more effective in acquiring certain skills (*n* = 8, 11.6%). A special advantage was mentioned considering experiential learning that can be achieved through VR, such as dementia training, which is otherwise difficult to simulate (*n* = 1, 1.4%).

### Disadvantages of VR (RQ7)

**Fig. 8:**
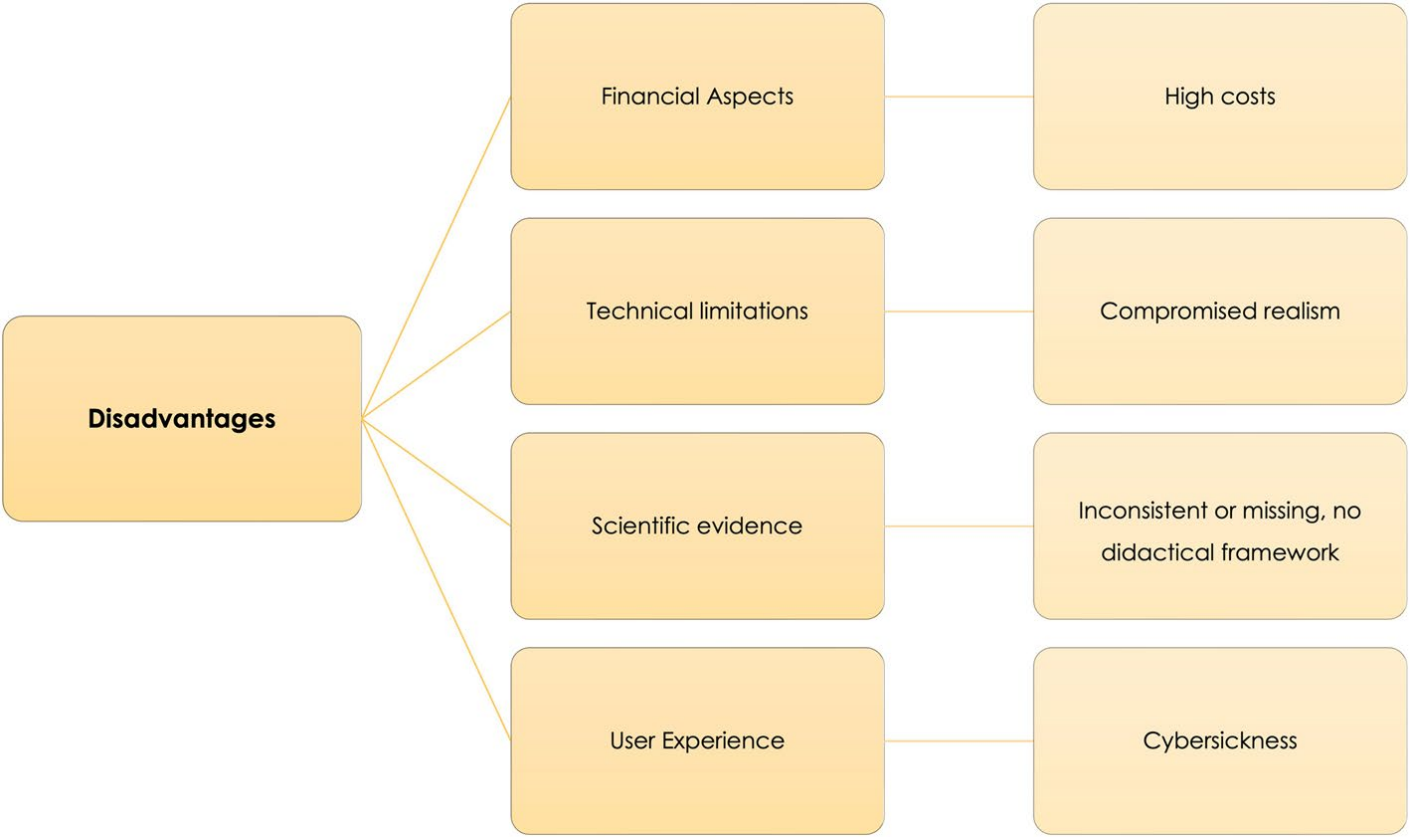
Most mentioned disadvantages of VR in medical education as reported in the included reviews

While VR has the potential to revolutionise medical education, it comes with several drawbacks that need to be considered. They can be categorised into (1) financial aspects, (2) technical limitations, (3) didactic and scientific evidence and (4) user experience (Fig. 8; Table 9).

**Table 9:** Disadvantages of VR simulations in medical education as reported by included reviews. Linked to RQ7.

REPORTED DISADVANTAGES	Number of Reviews	Review References
FINANCIAL ASPECTS		
High costs (time-intensive development and design requiring multidisciplinary team, acquisition of high-end hardware, personnel and technical support for maintenance, updates, training and administration).	30	[19, 29, 30, 34, 35, 37, 40, 41, 47, 48, 55, 59, 61, 63, 65, 66, 72, 84, 85, 92, 96, 97, 99–101, 104–107, 110]
**TECHNICAL LIMITATIONS**		
Limitations of how accurate real-life scenarios can be represented in VR (controllers instead of actual instruments, lack of realistic feedback like bleedings, variations in anatomy, tissue replication and deformation, depth perception in case of 2D simulations, complexity of human behaviour, reactions, traits and the diversity of population)	24	[29, 30, 35, 37–39, 41, 44, 48, 50, 58, 65, 73, 75, 84, 96, 98–100, 102, 104, 109, 112, 117]
Haptic feedback missing or unreliable (e.g., lack of force input, ergonomic limitations)	18	[29, 34, 35, 37, 38, 41, 48, 50, 65, 66, 68, 71, 75, 76, 84, 99, 100, 102]
**DIDACTIC ASPECTS AND SCIENTIFIC EVIDENCE**		
Inconsistent or missing evidence for the broad range of validity dimensions of acquired knowledge and skills, cost-effectiveness or cost-benefit-ratio due to the heterogeneity of objective study outcome measures and VR hardware. Missing theoretical learning framework as backbone of studies	24	[3, 11, 32, 36, 43, 44, 47, 60, 61, 63, 70, 75, 77, 84, 101, 103, 104, 107, 111, 113, 117]
Scenarios are often simplified, repetitive, highly specific with isolated tasks and not complemented with additional learning material, variation is limited	9	[34, 44, 47, 60, 64, 84, 100, 104, 107]
Lacking evidence for long-term retention of acquired knowledge and skills	5	[42, 44–46, 55]
Lack of multiplayer-scenarios including face-to-face communications and interconnection with team	5	[19, 34, 39, 47, 105]
Except for surgical simulators, most VR simulations focus on teaching cognitive skills, rarely on procedural or affective applications, lack of decision-making scenarios, consent processes, effective communication, leadership, i.e., non-technical skills	4	[39, 44, 48, 84]
Bias introduction in case of unfamiliarity with immersive VR and potential technical errors	4	[44, 64, 73, 75]
Unequal representation of medical specialties in VR applications	2	[3, 113]
Individual needs of lecturers not necessarily integrated in application design	1	[44]
**USER EXPERIENCE**		
Cybersickness in case of immersive VR using HMDs (e.g., motion sickness, nausea/vomiting, dizziness, cold sweats, asthenopia, fatigue, headache, neck discomfort, blurred vision, post-VR changes in static balance)	10	[34, 49, 50, 61, 66, 73, 85, 92, 105, 107]
VR simulations may induce overconfidence	1	[109]
Serious content of scenarios can cause stressful experiences, anxieties about performance or interpersonal dynamics	1	[61]
Potential of misuse, excessive use or game addiction	1	[61]

1. One of the biggest challenges relates to financial aspects, the most frequently cited in our literature research (*n* = 30, 43.5%). The development and design of VR applications for medical education require a multidisciplinary team, which is time-intensive and costly. Additionally, appropriate hardware, personnel and technical support for development and maintenance need to be acquired, users and teachers need to be trained and all these aspects are accompanied by an administrative outlay.
2. Despite high pace improvements, technical limitations are still an obstacle. Representing real-life scenarios accurately in VR can be difficult due to various factors, such as using controllers instead of actual instruments, a lack of realistic feedback (e.g., bleedings), missing variations in anatomy, tissue replication, and deformation, or depth perception in case of 2D simulations. Besides that, it is challenging to display authentic complexity of human behaviour, reactions and traits across diverse populations (*n* = 24, 34.7%). Particularly missing or unreliable haptic feedback (e.g., lack of force input, ergonomic limitations) was an often-mentioned disadvantage of VR training (*n* = 18, 26.1%).
3. When it comes to didactics and scientific evidence, inconsistent or missing evidence was reported for the broad range of validity dimensions of acquired knowledge and skills, cost-effectiveness, or cost-benefit-ratio due to the heterogeneity of objective study outcome measures, VR hardware and a missing theoretical learning framework as backbone of studies (*n* = 24, 34.7%). Furthermore, VR scenarios are often simplified, repetitive, and highly specific, with isolated tasks and not complemented with additional learning material, and accompanied by limited variations (*n* = 9, 13.0%). Currently, there is still a lack of evidence for long-term retention of acquired knowledge and skills. Apart from surgical simulators, most VR simulations focus on teaching cognitive skills, with few procedural or affective applications. They lack decision-making scenarios, consent processes, effective communication, as well as training of e.g., leadership, which especially features NTS (*n* = 4, 5.8%).
4. Further challenges are related to the user experience. Cybersickness, such as motion sickness, nausea, dizziness, cold sweats, asthenopia, fatigue, headache, neck discomfort, blurred vision, and changes in static balance form significant concerns for immersive VR using HMDs (*n* = 10, 14.5%). One review respectively (1.4%) mentioned that VR simulations may also induce overconfidence or have potential for misuse, excessive use, or game addiction.

#### Mitigation strategies

Considering the mentioned disadvantages of VR, some reviews reported possible means of mitigation: costs of hard- and software tend to decrease over time [34, 35], and cost-effectiveness could be achieved in the long-term [16, 36, 37]. Technological advancements could make up for missing haptic feedback [19, 38, 39], e.g., by using devices such as gloves [19]. Future research of interdisciplinary teams can help to provide standardised systematic guidelines [40] for comparisons at different levels of validity and structured curriculum implementation [41] enables comparisons at larger scale for a higher level of evidence [3, 42], circumventing heterogeneity of studies and their outcomes [43]. Further investigations on long-term retention of gained knowledge and skills in VR training scenarios are recommended since the evidence base is still lacking [39, 41, 44, 45]. This also includes exploring the necessity of refreshing training sessions [46, 47] and to critically rethink the use of multiple-choice questionnaires or pure test scores for the assessment of knowledge gain and depth of understanding [38, 44]. Advancements in Natural-Language-Processing and AI are necessary [48] to create more interactive and realistic training scenarios. Cybersickness can be tackled by reduced latency and increased frames per second [34, 49], as well as the use of fixed backgrounds [50] or movement sensors [49].

## Discussion

This scoping review summarises findings of a reproducible systematic literature research in March 2022 with a total of 69 included reviews covering a broad range of VR application fields in medical education. Research questions focused on technical and didactical requirements for the implementation of VR courses, evaluation methods and results, as well as reported advantages and disadvantages of using VR as a medical education tool. To ensure highest reliability with a broad and as complete, current, and precise as possible synopsis, we solely focused on reviews including studies between 2012 and 2022 and added current publications in the discussion.

### Characteristics of included reviews

In the last decade, an enhancing relevance of VR in medical education is shown by an increasing number of reviews and studies since 2012. Hotspots are still high-income countries, while low-income countries are rather underrepresented. For example, the fact that we found no review originating from Africa indicates that VR technology by now has not yet become routine, particularly not in low-income countries, which is in agreement with previous findings [3, 19, 51].

Concerning specialties that use VR for educational purposes, we can approve findings of other reviews which identified surgical use cases to be predominant [19, 52, 53] - especially minimally invasive types (laparoscopic, endoscopic) - with a clear focus on procedural skills, followed by emergency medicine scenarios. The background reason for implementing VR training in these subjects is most likely linked to one of the biggest advantages reported for this technology: the repeatability of procedures without causing any harm or danger to patients and/or practitioners. By acquiring routine in a safe environment with realistic conditions, using VR courses enhances preparation for real life situations where clinical management is based on algorithms and requires fast decision-making. Even home-based training sessions might be feasible [19, 54]. Furthermore, the predominance of both surgical use cases and physicians as study participants reported by the included reviews indicates that VR has mainly been investigated and used regarding advanced medical and clinical training instead of basic education of undergraduate medical [51] or nursing students [55].

### Technical requirements

According to the high rate of surgical VR applications, realistic haptic feedback and accurate interaction regarding tissues were mentioned as technical requirements. Naturally, manual skills required for surgical disciplines are easier to be embedded in simulations compared to other competencies such as NTS which demand multiplayer modes or highly intelligent virtual agents. Although the technical advancements already enable extending the range of training scenarios accordingly [19], they are still underrepresented as reflected in our review. Technical issues and barriers must be resolved before the VR application is regularly used to guarantee that they are not negatively affecting the learning process and user experience [20].

### Type of VR

Only 11 out of 69 included studies focused on immersive forms of VR, i.e., for which e.g., HMDs are required in contrast to non or less immersive forms such as screen-based applications. This broad conceptual perception and definition of VR is nowadays increasingly shifting towards immersive VR with HMDs [56] due to the technological advancements: increasingly realistic VR training scenarios with higher feeling of situational presence. However - since widespread usage of the technology is still limited - available publications have addressed this form of VR only rarely. The required degree of realism which is serving the learning goals deserves further investigation as well [57].

### Didactical requirements

Remarkably, a majority of reviews indicate the need for integration of profound learning theory concepts into respective applications [44] taking into account different levels of learners’ experience [46]. The development of standardised guidelines for evaluation and integration into medical curricula has been largely lacking up to now [15, 51, 54, 56, 57] and might be impeded by deviating degrees of pretraining familiarity of participants with VR. Emblematic of this, the included reviews hardly contained any information on these specific requirements and almost 40% did not cover requirements at all.

Concurrently, innovations in medical curricula implementing VR must address how to avoid that users receive incorrect automatically generated evaluation feedback through the application and how to identify responsibilities in case of incorrect feedback [58]. Furthermore, it must be discussed how to prevent VR from being maliciously used or causing any decrease of valuable and necessary personal interactions between teachers and learners, also regarding evaluation feedback within or outside of VR applications [58]. For example, lower performance of study participants after the simulation was observed when no external feedback from experts was provided in addition to the automatically generated feedback through the application [59]. Thus, a hybrid form of evaluation feedback within and outside of the application might be preferable [48] and should be subject of further research [57].

VR can only be useful and effective if it is used in a reasonable way supported by a profound theoretical backbone to prevent from negative effects on the established curricula [44, 60]. Thus, VR scenarios in the form of serious games can take advantage of intrinsic and extrinsic motivation by gamification [61, 62].

Therefore, it is strongly recommended to not consider VR as a completely independent stand-alone solution replacing already established learning formats but to consider it as a potential additionally supporting tool to existing learning programmes [17, 35, 51, 60, 63–67] closing gaps of traditional methods.

### Study methodologies and outcomes

The considered reviews clearly indicated the need to check all levels of validity [68] for VR applications, especially transfer validity, and to demonstrate their efficacy which has not been investigated to a sufficient level to date. Further research and controlled trials are required to validate tools considering these aspects. For example, complication rates should comprise postoperative complications besides intraoperative complications [69].

The mixed results reported in some studies can be linked to the already reported missing guidelines for both effective study design, i.e. randomised-controlled trials with large sample sizes [49, 55, 57, 67], and lacking standardised study evaluation measures, e.g. by a combination of subjective (e.g. by experts) and objective, discriminant and reproducible validation assessments [36, 63, 70–72], quantitative and qualitative data [46] and unified definition of effectiveness criteria [73] for large scale comparisons [56]. This includes aspects such as user experience, immersion, cybersickness, cognitive load and ascending levels of validity and is linked to several sets of variables: individual learner, learning environment, context, technology, and pedagogy [73, 74]. Though, considering comparisons, participant blindness can be hardly achieved due to the nature of VR applications [75].

In addition, long-term retention of acquired knowledge and skills have not been covered sufficiently in studies to date [44, 46]. Patient benefit and quality of care are strongly recommended as ultimate objectives of testing procedures, but often remain untested [56, 76, 77]. Furthermore, cost-effectiveness should be kept in mind throughout the whole developmental process and finally needs evaluation [3, 46, 47, 70, 73]. It is recommended to draft respective guidelines based upon and/or adapted from existing or new frameworks, e.g., considering VR treatments in patient care [78], digital health interventions [79], e-learning [74] and shared experiences [80]. Other sources could be Kirkpatrick’s evaluation framework [52] or Blooms’ taxonomy of educational objectives comprising cognitive, procedural and affective learning domains [81] amongst others [82].

### Advantages of VR

Our findings indicate that VR technology is increasingly accessible and convenient, even for lower-income countries using low-cost models [83]. It offers advantages such as flexibility in terms of time and space, repeatability of training, and cost-effectiveness, which is also linked to the choice of high-fidelity or low-fidelity simulations [30].

“Transfer Effectiveness Ratio” is the only validated measure of cost-effectiveness and should be used in further research on VR used for training. This ratio quantifies the difference between VR and real life in terms of the time (and cost) required to achieve fully competent performance [30, 36].

While providing a safe and controlled environment for learning, VR simulations allow users to make mistakes and training without time constraints [84]. The variability of VR scenarios enables to practise both skills related to basic tasks and complex procedures, theoretical knowledge and NTS [73], including unexpected or low frequency scenarios as well as high-pressure situations. This makes it interesting for both low-experienced users and high-experienced users aiming to improve complex skills and situations [85, 86]. Zhang et al. [84] reported that VR simulator training has been shown to be comparable to clinical training with similar outcomes. VR technology could enable patient-specific simulations [41], or enhance pre-operative planning [29, 87, 88], be adapted to the individual learning preferences and levels of knowledge of the user [42], or even suggest next training steps based upon advanced algorithms [38].

VR enhances training authenticity, helps develop spatial understanding and visualisation skills [43, 44], and can offer multi-user scenarios that promote soft skills such as teamwork [11, 89]. This goes along with the increasing focus on NTS in medical curricula. Advancing AI technology expands the use cases of VR as a psychomotor training tool. Complex scenarios and real time speech recognition foster decision-making, situational awareness, and communication skills. Flin et al. [90] define NTS as cognitive, social, and personal resource skills complementing technical skills and contributing to safe and efficient task performance. According to them, NTS include individual cognitive skills (e.g., situation awareness, decision-making, coping with stress, and management of fatigue) and interprofessional social skills (e.g., cooperation and teamwork, conflict resolution, leadership, empathy) [90]. The importance of these competencies is recognised by currently revised learning goals, e.g., in German medical schools [91] and should be examined in more detail as part of future research and development [30].

According to Tang et al. [92], using immersive technology for medical education not only facilitates illustrating complex ideas while removing geographical constraints, but also eases data collection for later performance assessments and evaluation feedback.

### Disadvantages of VR

Despite its potential to eminently shape future medical education, VR still comes with several considerable drawbacks. Although prices for hardware are decreasing, financial aspects remain a significant challenge, as to date VR development requires a multidisciplinary team, high-performance hardware, and concrete course-integrations demand technical support, along with training for users and teachers. The multidisciplinary team and the inclusion of end users, such as students and lecturers in the development process can help to successfully implement VR in medical education and contribute to the motivation for higher use and acceptance of VR solutions [20], also by considering the individual needs of lecturers and targeted learning outcomes [64, 93].

Furthermore, technical limitations, such as difficulty in accurately representing real-life scenarios, lack of realistic feedback, and challenges in displaying authentic human behaviour, pose obstacles to VR training. Nevertheless, Trehan et al. [65] stated, that “simulation is not meant to eliminate the need for genuine patient interaction and real operating room (OR) experience, but to serve as an important adjunct for safer transition to independent patient care and continued practice”.

Didactically, inconsistent or missing evidence considering long-term retention of knowledge and skills, as well as a lack of theoretical frameworks hamper the assessment of competences acquired through VR [19]. A good example of severe necessity for VR training concepts is shown by the fact that since 2018 access to the operating room (OR) has been permitted without prior simulation training in some instances [42]. Madan et al. [37] suggest combining the strengths of different simulation methods to compensate for their individual weaknesses while providing added value for skill acquisition. At the same time, they emphasise that simulations can only cover a portion of the whole learning while the actual patient must remain as “the final teacher”.

In addition, user experience aspects require to be approached, e.g., including cybersickness [85] and technical problems which could strongly affect the user’s satisfaction [64, 75, 94].

#### Mitigation strategies

The included reviews have already presented some potential solutions for existing drawbacks. However, we can think of further means for mitigation: some of them addressable by technological advancements and AI, e.g., higher degree of realism and representation of real-life variations, more multiplayer-scenarios and intelligent virtual agents to sufficiently train NTS. Variability and completeness of complex training scenarios, including accompanying learning material and curriculum integration could be achieved by future research with interdisciplinary development teams stemming from a university context and thus having direct influence on medical curricula. Careful planning of tutorial sessions or free navigation periods in VR [46] could make up for any novice-bias caused by less familiarity with VR, especially in comparative studies with traditional learning methods [44] and might shorten learning curves in VR simulations. Tutorial sessions could also compensate for divergent perceptions of VR-naive and VR-experienced users prior to training scenarios [70] and deserves future investigation [94]. At the same time, it was observed that learners with less experience could be even more easily motivated by fictional scenarios [53, 85].

The still unequal representation of medical specialties and corresponding training scenarios might be addressed by prospective research projects expanding the fields of possible applications. Misleading overconfidence after VR simulations [53] could be prevented by the development of more objective measures and respective evaluation feedback. Excessive use, misuse or game addiction could be counteracted by careful application design and consideration of addiction prevention strategies. One review pointed out that the serious content of VR training scenarios could cause stressful experiences and anxieties [61]. However, we argue that the same risks can apply for other forms of training scenarios and are even a desired effect since they reflect context and emotions of real-world settings.

It could be beneficial to further explore gender-specific phenomena, e.g., female participants were reported to be more likely to be affected by cybersickness [73] while male showed a tendency for better performance and higher interest regarding laparoscopic simulations [37].

The increasing amount of VR content might offer the opportunity to develop overarching, universal platforms for sharing and making solutions globally available for health care education [19, 51, 72]. This could include shared case libraries [48, 56] as well as providing applications in multiple languages [56] and may reduce barriers such as acceptance and assist with the implementation in health care education [72]. Though, such a unification and open-source policy is obviously accompanied by divergent financial interests and lack of compatibility between systems.

### Strengths and limitations of this review

We have considered in this review all kinds of health care professionals’ education and training without restriction to medical students only. A comprehensive literature search strategy was applied in five scientific literature databases. Screening and data extraction were independently conducted by two researchers in parallel and discussed in case of conflicting decisions to increase soundness while decreasing any subjective bias. Following the established methodology, this scoping review does not include an assessment of quality of evidence. Our search was limited to reviews published in English and German. The description of evaluation study designs in the included reviews is provided in an exemplary way in this review but not to full extent, since we focused on the evaluation target measures and results rather than their methods. Additionally, some studies might have been included by more than one of the inspected reviews. Definitions and perceptions of VR have changed over time [95], making comparisons of results partially difficult if the definition has not been clearly stated in the publication. All considered publications were peer-reviewed publications of the article type “review”. Thus, some other relevant publications, conference papers, books, etc. might have been overlooked. To ensure actuality, we have conducted additional literature research before the submission of this publication and referenced new appropriate findings.

## Conclusions

VR in medical education offers a multitude of benefits that span various categories. The technology’s practicality, user experience, and skill development potential make it an effective tool in medical education. Furthermore, the versatility of VR in terms of content, assessment, and evaluation feedback, as well as unique didactic advantages make it a valuable asset in medical education today that ultimately seems to improve patient safety and outcome. Currently observed disadvantages concerning financial aspects, technical limitations, didactic aspects and lacking scientific evidence, as well as user experience issues can be addressed by multidisciplinary collaborations in research and development for which this review can provide crucial indications and recommendations.

This review outlined the evolution and the current state of where and how VR technology is rooted in medical education and pointed out the strong need for standardised and validated guidelines for research, evaluation, didactic frameworks, and development projects to guarantee high quality of evidence. Alongside, multidisciplinary collaborations between physicians, medical didactics experts, 3D-designers, VR software developers as well as AI-experts and the end users are needed to create scientifically sound backbones for the development and integration of useful VR applications in medical education.

## Data Availability

and other materials. Bibliographic files and data extracted from included studies are available from the authors upon reasonable request.

## Abbreviations

VR: Virtual Reality
AI: Artificial Intelligence
NTS: Non-technical skills
OR: Operating room
OSACSS: Objective Structured Assessment of Cataract Surgical Skill
